# Capecitabine-induced-coronary-vasospasm leading to polymorphic ventricular tachycardia and cardiac arrest

**DOI:** 10.1186/s40959-024-00214-4

**Published:** 2024-02-27

**Authors:** Maciej Kabat, Roma Padalkar, Sara Hazaveh, Vladimir Joseph, David Feigenblum, Sean Sadikot

**Affiliations:** 1 Department of Internal Medicine, Hackensack University Medical Center, 30 Prospect Avenue, Hackensack, NJ 07601 USA; 2 Department of Critical Care, Hackensack University Medical Center, 30 Prospect Avenue, Hackensack, NJ 07601 USA; 3 Department of Cardiology, Hackensack University Medical Center, 30 Prospect Avenue, Hackensack, NJ 07601 USA; 4 Department of Cardiac Electrophysiology, Hackensack University Medical Center, 30 Prospect Avenue, Hackensack, NJ 07601 USA

**Keywords:** Capecitabine, Coronary vasospasm, Polymorphic ventricular tachycardia, Colorectal carcinoma

## Abstract

Capecitabine, a pro-drug of 5-fluorouracil, is commonly used in the treatment of breast and colorectal cancer. Its side effects, including nausea, vomiting, diarrhea, fatigue, loss of appetite, and bone marrow suppression, are well recognized. However, coronary vasospasm represents a less commonly recognized but significant complication of fluoropyrimidine-based therapies such as capecitabine. Proposed mechanisms for this adverse effect complication include direct endothelium-independent vasoconstriction, activation of protein kinase C, and activation of the cyclooxygenase pathway. In this report, we present a case of capecitabine-induced coronary vasospasm leading to progressive, focal ST-elevations, myocardial ischemia, and subsequently polymorphic ventricular tachycardia. These events were captured on telemetry, in a male in his early 40s, diagnosed with stage IIIB sigmoid colon cancer. Notably, the patient had no pre-existing coronary artery disease or other cardiovascular risk factors. Upon diagnosis, the patient was initiated on a calcium channel blocker, verapamil, to mitigate further coronary vasospasm events. After thorough discussions that prioritized the patient’s input and values, an implantable cardioverter-defibrillator was placed subcutaneously. Following discharge, the patient restarted capecitabine therapy along with verapamil prophylaxis and did not experience any subsequent shocks from his ICD as assessed during his outpatient follow-up visits. This case emphasizes the need to involve patients in decision-making processes, especially when managing unexpected and serious complications, to ensure treatments align with their quality of life and personal preferences.

## Introduction

Capecitabine, a fluoropyrimidine class oral chemotherapy drug, is predominantly used for the treatment of breast and colorectal cancers. This pro-drug undergoes metabolic conversion by the enzyme thymidine phosphorylase, expressed at higher levels within tumor cells, into its active counterpart, 5-fluorouracil (5-FU). This process interferes with DNA synthesis, effectively inhibiting cancer cell proliferation and leading to tumor cell death. The dosage and duration of capecitabine therapy are tailored according to the type and stage of cancer, as well as the specific circumstances of each patient. This fluoropyrimidine class is known to cause several side effects, such as nausea, vomiting, diarrhea, hand-foot syndrome, fatigue, loss of appetite, hair loss, and bone marrow suppression.

However, cardiotoxicity represents a significant but less acknowledged complication of fluoropyrimidine-based therapies, including capecitabine. The reported incidence of cardiotoxicity ranges from 1 to 35%, depending on the specific criteria used to define cardiotoxicity, with some studies classifying bradycardia under this category, the demographic of the patient group, the type of fluoropyrimidine therapy, and the method of administration [1–8]. The most common manifestation of cardiotoxicity is chest pain, both typical and atypical, but patients may also experience arrhythmias, hypotension, coronary vasospasms, pericarditis, stress cardiomyopathy, myocarditis, and even cardiac arrest [1, 9]. The initial side effects typically emerge around 12 h post-administration, although they may appear several days following treatment [10]. Risk factors that increase the likelihood of cardiotoxic effects include extended duration of therapy, existing coronary or structural heart disease, chronic kidney disease, and the use of other cardiotoxic medications. Nevertheless, cardiotoxicity can still pose a threat to individuals lacking these predisposing conditions, underscoring the need for vigilant monitoring and management in all patients undergoing fluoropyrimidine-based therapy [1]. 

The cardiotoxic effects of fluoropyrimidines, such as capecitabine, are supported by extensive research from both in-vitro and clinical studies [11, 12]. These mechanisms are broadly classified into two general types: direct and indirect myocardial toxicity [13]. Direct myocardial toxicity involves direct harm to the myocardial tissue. The active metabolite of capecitabine, 5-FU, undergoes further biotransformation into alpha-fluoro-beta-hydroxypropionic acid and fluoroacetate, both of which exhibit direct cardiotoxic effects on the myocardium [14]. Additionally, studies have reported that 5-FU can damage the coronary artery endothelium, leading to distal coronary thrombosis and myocarditis [15, 16]. Indirectly, fluoropyrimidines are implicated in causing coronary artery vasospasm by multiple theorized mechanisms: direct endothelium-independent vasoconstriction, activation of protein kinase C, and activation of the cyclooxygenase pathway [11]. 

Coronary vasospasm is considered the predominant mechanism underlying chest pain and myocardial ischemia among patients undergoing fluoropyrimidine therapy [1, 17]. The frequency of coronary vasospasm reported in patients receiving such treatments varies significantly. For instance, one study observed that approximately 2% of patients treated with a fluoropyrimidine therapy experienced vasospasm [3]. In contrast, other research indicates that the incidence could be as high as 13% [18]. Interestingly, patients who developed vasospasm were younger and had fewer cardiovascular risk factors. Furthermore, these individuals were less likely to be receiving prophylactic treatment for vasospasm, such as a calcium-channel blocker, suggesting a potential area for intervention to reduce the risk of this complication [3, 19]. 

The prevailing approach to managing fluoropyrimidine-associated chest pain promptly involves halting the medication and applying a combination of nitrates and calcium channel blockers. However, rechallenging the fluoropyrimidine may be deemed necessary from an oncological perspective. Despite this, there is a lack of consensus and guidelines on the prophylactic use of these agents to reduce the incidence of fluoropyrimidine-associated cardiotoxicity [20]. Historical data reveal that attempting to rechallenge patients with fluoropyrimidines leads to a recurrence rate of cardiotoxicity up to 90% and a mortality rate as high as 13%, even with the use of antispasmodic agents [20–22]. In the absence of significant changes in guidelines over recent years, alternative approaches have been explored including discontinuing the fluoropyrimidine treatment, opting for a non-fluoropyrimidine regimen, reducing the dosage, and attempting retreatment with the initial medication, and transitioning from infusional to bolus 5-FU administration to lower the risk of toxicity from accumulated metabolites. Additionally, the integration of nitrates and calcium channel blockers has become a standard component in every rechallenge protocol. Recent studies have shown success in rechallenging patients with the initial offending drug [23–27]. Decisions regarding rechallenging a patient need to involve a comprehensive discussion among cardiology, oncology, and the patient. The ultimate objective remains to deliver the most efficacious treatment while minimizing potential toxic side effects.

This report describes a case of capecitabine-induced coronary vasospasm leading to progressive, focal ST-elevations, myocardial ischemia, and subsequently polymorphic ventricular tachycardia. These events were captured on telemetry, in a male in his early 40s, diagnosed with stage IIIB sigmoid colon cancer. Notably, the patient had no pre-existing coronary artery disease or other cardiovascular risk factors. Upon diagnosis, the patient was initiated on a calcium channel blocker, verapamil, to mitigate further coronary vasospasm events. Given that the patient experienced a cardiac arrest requiring cardiopulmonary resuscitation and multiple defibrillator shocks, a collaborative decision-making process with the patient led to the placement of a subcutaneous implantable cardioverter-defibrillator (ICD). This case emphasizes the profound impact of unexpected cardiotoxic side effects of capecitabine on our patient’s life, highlighting the importance of shared decision-making discussions and the thorough consideration of each patient’s quality of life in medical decision-making.

## Case presentation

**Fig. 1:**
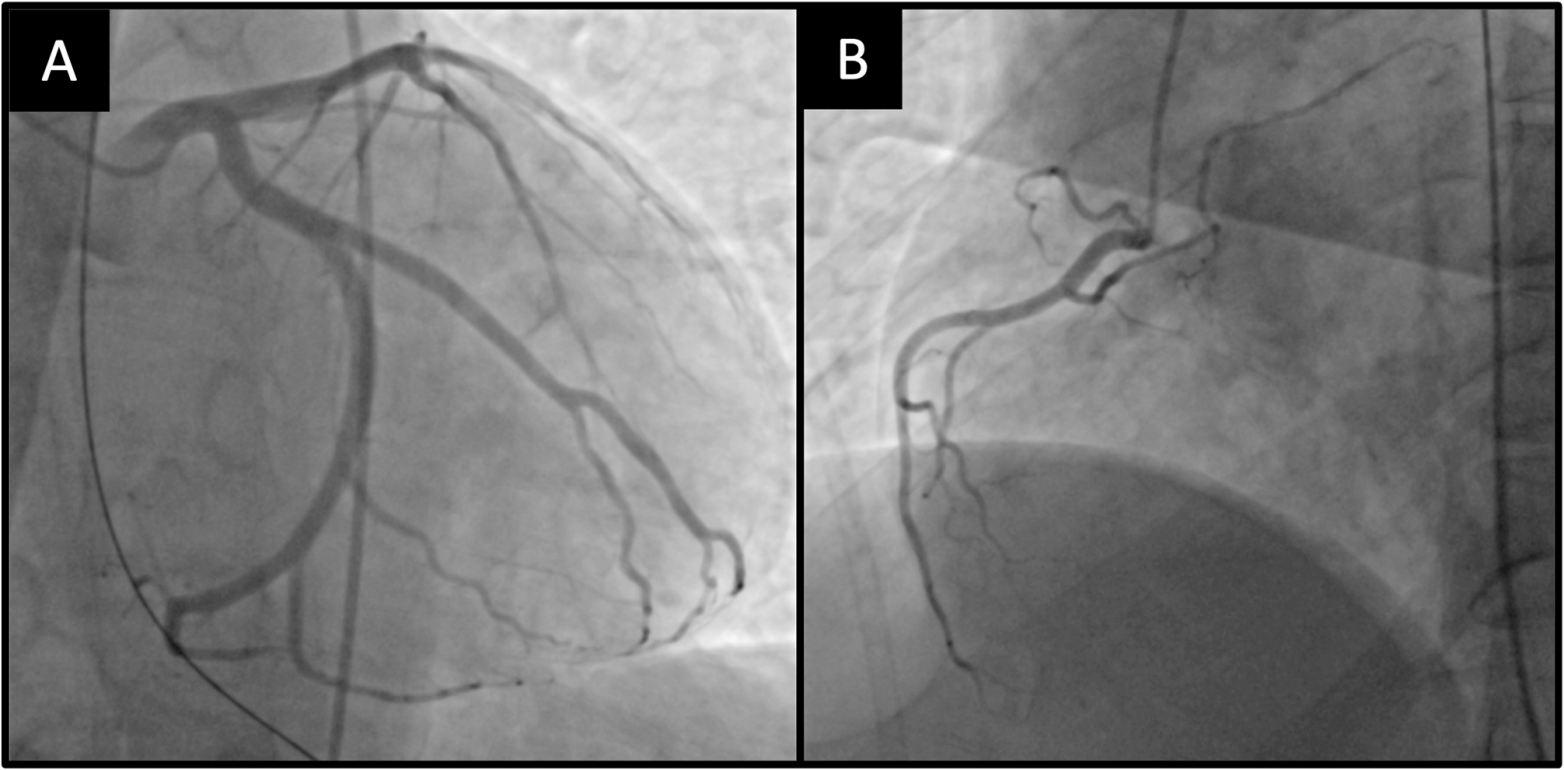
Coronary Angiogram. Left coronary angiogram (**A**) and right coronary angiogram (**B**) without any significant coronary artery disease. Left-sided dominant circulation

A male in his early 40s, diagnosed with stage IIIB sigmoid colon cancer underwent hemicolectomy and was initiated on adjuvant chemotherapy with oxaliplatin and capecitabine one month prior to presenting to the emergency department after experiencing cardiac arrest which occurred while running in the park. Approximately three days prior to the cardiac arrest, the patient developed exertional chest pressure and shortness of breath. He did not seek medical attention at that time. As an avid runner, the patient had no personal or family history of cardiac disease. The cardiac arrest occurred suddenly during his first attempt at resuming his normal running routine since his surgical procedure. Bystanders initiated cardiopulmonary resuscitation, and emergency medical services were immediately called. The patient received two shocks with an automated external defibrillator before achieving return of spontaneous circulation. He was intubated in the field, transferred to our institution, and initiated on hypothermia protocol (targeted temperature of 37.5℃ or less) [28]. The electrocardiogram (ECG) showed sinus rhythm without ischemic changes, and all waves and intervals were within normal limits. A CT pulmonary angiography scan noted a right lower lobe parenchymal infiltrates consistent with aspiration during the cardiac arrest. Troponin-I level was found to be elevated to 1.45 ng/mL (reference range 0.0-0.02ng/mL). An echocardiogram performed on the same day revealed normal left ventricular size and thickness, normal systolic and diastolic function, and no significant valvular pathology. Due to suspicion of ventricular tachycardia or fibrillation arrest secondary to myocardial infarction, the patient underwent cardiac catheterization which revealed no significant coronary artery disease with left-sided dominant circulation (Fig. 1).

**Fig. 2:**
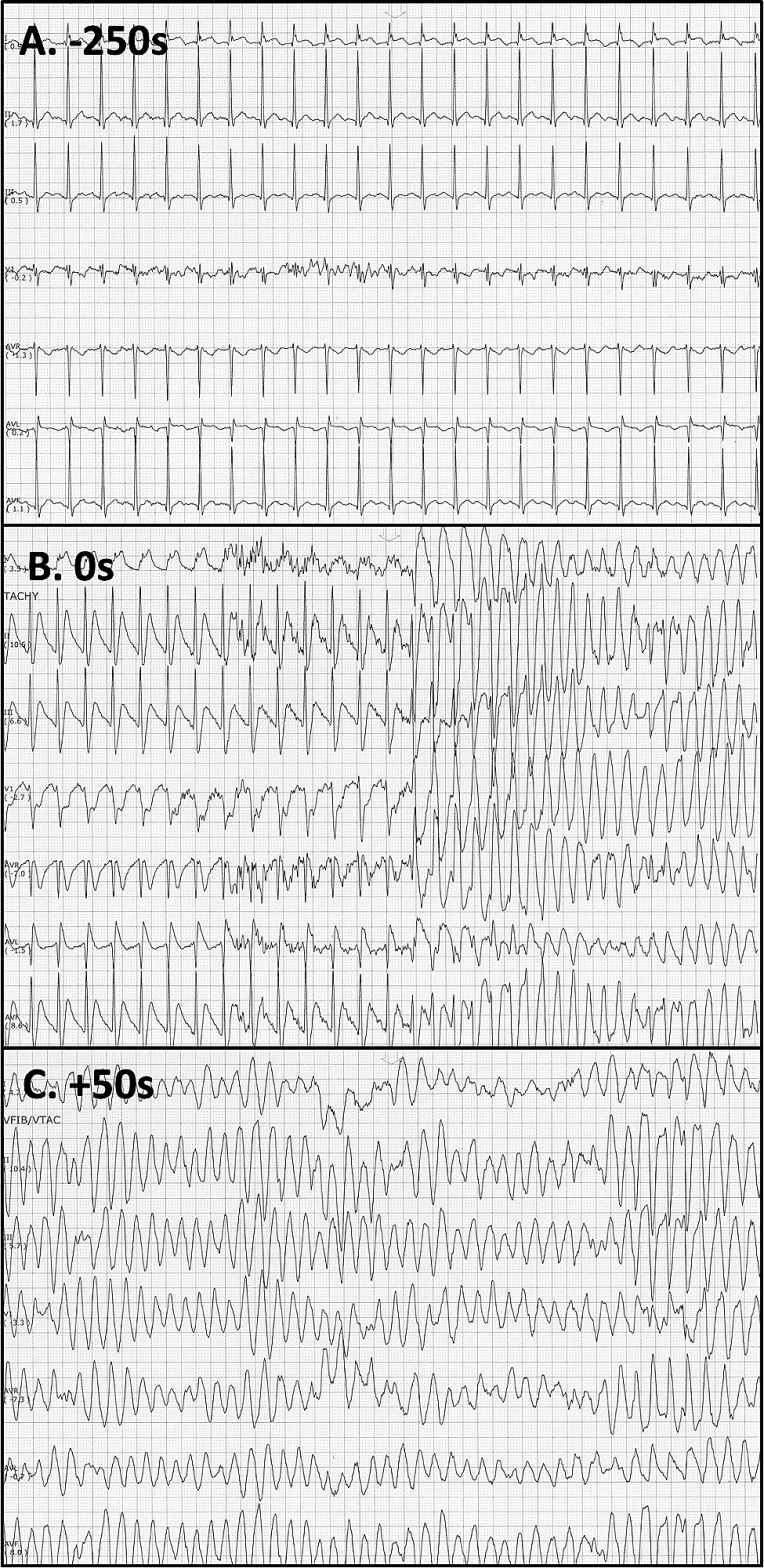
Telemetry. The time on onset of the PVT is 0 s. Progressive tachycardia is seen at about − 250 s (**A**) with focal ST-segment elevation and reciprocal ST depressions were observed at -150 to 0 s (**B**) before the onset of the arrhythmia (**C**). There was no prolonged QT interval noted on telemetry

The following day, the patient became febrile and tachycardic with a temperature of 102.9 °F. He subsequently experienced an episode of polymorphic ventricular arrhythmia (PVT), as detailed in Fig. 2. The patient’s pulses were maintained, and the episode spontaneously resolved after approximately 150 s. All the patient’s electrolytes were within normal limits, including a magnesium level of 2.25 mg/dL (Reference 1.6–2.6 mg/dL). After reviewing telemetry, as seen in Fig. 2, focal ST elevations with reciprocal ST depressions were observed starting 150 s before the onset of the arrhythmia. There was no prolonged QT interval noted on telemetry. A cardiac MRI revealed normal biventricular function and no late gadolinium enhancement indicative of myocardial fibrosis, myocarditis, or myocardial edema. Given the normal cardiac MRI and non-obstructive coronary disease, the patient was diagnosed with PVT resulting from coronary vasospasm induced by capecitabine. Consequently, capecitabine was promptly discontinued and the patient was initiated on a calcium channel blocker, verapamil, to prevent coronary vasospasm and to maintain a heart rate below 80 beats per minute to prevent the development of arrhythmias. After shared decision-making discussions with the patient, the decision was made to place a subcutaneous implantable cardioverter-defibrillator (ICD), and the patient was discharged with verapamil therapy and close follow-up from cardiology, electrophysiology, and oncology. After discharge, while on verapamil, he underwent an exercise stress test, which was within normal limits, further ruling out catecholaminergic arrhythmia. An arrhythmia comprehensive panel test for the deletion/duplication of 41 genes was performed and yielded negative results, ruling out any genetic cause of arrhythmia. After clearance from cardiology, the patient restarted capecitabine therapy along with verapamil prophylaxis. Fortunately, the patient did not experience any subsequent episodes of chest discomfort, angina, or palpitations. The ICD was also interrogated which did not reveal any evidence of arrhythmia nor deliver shocks. The patient currently remains symptom-free from a cardiology standpoint and is gradually increasing the amount of running at his own pace.

## Patient perspective

The impact of capecitabine-induced coronary vasospasm and subsequent cardiac arrest on this patient’s life cannot be overstated. For our patient in his early 40s, running was not merely a hobby but a vital part of his identity and overall well-being. Running provided him with physical and emotional relief, offering a sense of control amid the challenging fight with cancer. Additionally, his chemotherapy, which included capecitabine, played a critical role in his battle against rectal cancer, offering hope for a future free from the disease.

However, the sudden and life-threatening cardiac arrest that he experienced drastically altered his life. It not only disrupted his physical health but also inflicted profound psychological trauma. The fear of sudden cardiac arrest, something previously unimaginable, now became a concern in an activity he once cherished. The intersection of his passion for running and the necessity of chemotherapy presented a significant threat to his health.

Extensive discussions among critical care, electrophysiology, cardiology, and oncology teams focused on various treatment options. Prior to this hospitalization, the patient chose the oxaliplatin plus oral capecitabine-based chemotherapy for its shorter course and the convenience of oral therapy. The alternative regimen, oxaliplatin plus leucovorin and short infusional 5-FU, required an ambulatory infusion pump, which the patient did not value. Additionally, 5-FU provided similar theoretical vasospasm risks to capecitabine.

The patient decisively chose to proceed with capecitabine-based chemotherapy for the goal of at least six months as outlined by his oncologist. Preferring to prioritize options that minimize risks while resuming capecitabine, he was well aware that the prophylactic use of calcium channel blockers and nitrates might not definitively prevent coronary vasospasm. He viewed the ICD as a solution to alleviate the anxiety and fear of another cardiac arrest, thereby allowing him to complete his chemotherapy regimen while maintaining an active lifestyle which included running. He recognized the potential for calcium-channel blockers to reduce the risk of vasospasm but, having endured a life-threatening event and its aftermath, he was naturally hesitant to resume fluoropyrimidine therapy without a protective measure against possible future cardiac incidents. Opting for an ICD as a form of secondary prevention offered the reassurance needed to balance his treatment needs and his aspirations for quality of life and adherence to personal values.

## Discussion

In the report above, we detail the experience of a male in his early 40s, diagnosed with stage IIIB sigmoid colon cancer, who began adjuvant chemotherapy with oxaliplatin and capecitabine one month before he suffered a cardiac arrest while jogging in the park. Immediate cardiopulmonary resuscitation and two shocks from an automated external defibrillator were required to restore spontaneous circulation. Subsequent left cardiac catheterization revealed no significant coronary artery disease. Despite being an avid runner, which could predispose him to exercise-induced PVT, the recurrence of an episode while at rest, coupled with a later normal exercise stress test, pointed towards alternative etiologies [29]. Additionally, negative genetic testing further diminished the likelihood of hereditary heart conditions. ICU telemetry captured transient focal ST-segment elevation with reciprocal ST-depression changes, which normalized post-episode. These observations, combined with the absence of any other causes for increased myocardial oxygen demand, led to the diagnosis of capecitabine-induced coronary vasospasm, resulting in PVT and cardiac arrest.

Clinical evidence has shown that coronary vasospasm can be precipitated by a variety of factors including alterations in autonomic activity, the use of beta-blockers, smoking, catecholamine release, the consumption of recreational drugs, exposure to chemotherapy agents, hyperventilation, exposure to extreme cold, and physical and/or mental stress [30–34]. In the case above, our patient experienced two distinct episodes of coronary vasospasm: one during physical exertion and another while in the ICU, coinciding with a febrile state. While exercise alone is not typically an independent cause of coronary vasospasm, there are documented instances where increased catecholamine levels from physical activity have triggered such events [34–37]. The standard management for coronary vasospasm associated with fluoropyrimidine treatment involves stopping the causative drug and administering a regimen of nitrates and calcium channel blockers. Nonetheless, rechallenging the same chemotherapy regimen may be deemed necessary from an oncological perspective. This raises the question of whether early intervention with a calcium channel blocker upon the onset of chest pain could have averted the subsequent cardiac arrest. Following the patient’s ICU admission, oncology advised rechallenging with capecitabine as the optimal approach to address his cancer, once his condition stabilized and it was deemed safe to proceed. The potential efficacy of prophylactic verapamil and sublingual nitroglycerine in preventing further cardiac episodes was discussed with the patient. After thorough discussions among the multidisciplinary team, the patient expressed a clear choice for the installation of a permanent subcutaneous ICD before hospital discharge, influenced by the prior cardiac arrest experience. Following discharge, the patient restarted capecitabine therapy along with verapamil prophylaxis and experienced no subsequent episodes of chest discomfort, angina, or palpitations. The absence of shocks administered by the ICD further confirmed the effectiveness of this approach. This outcome suggests that the ICD might not have been necessary; however, given the circumstances, not taking such a precaution was a risk the patient was unwilling to accept.

This case underscores the challenges in balancing cancer treatment efficacy with cardiac health and overall well-being. It highlights the importance of recognizing and addressing the emotional toll and anxiety that adverse drug reactions can cause. Healthcare providers play a crucial role in educating patients about potential risks and side effects and in creating strategies to mitigate these risks. Ultimately, this serves as a reminder that medical decisions must consider the profound impact they have on individuals and their overall quality of life.

## Data Availability

The authors can confirm that all relevant data are included in the article.

## Abbreviations

5-FU: 5-fluorouracil
PVT: Polymorphic ventricular tachycardia
CT: Computed tomography
ECG: Electrocardiogram
ICD: Implantable cardioverter defibrillator
